# Changes in cancer screening before and during COVID‐19: findings from the Korean National Cancer Screening Survey 2019 and 2020

**DOI:** 10.4178/epih.e2022051

**Published:** 2022-05-30

**Authors:** Thao Thi Kim Trinh, Yun Yeong Lee, Mina Suh, Jae Kwan Jun, Kui Son Choi

**Affiliations:** 1 Graduate School of Cancer Science and Policy, National Cancer Center, Goyang, Korea; 2 National Cancer Control Institute, National Cancer Center, Goyang, Korea

**Keywords:** Neoplasms, Mass screening, COVID-19

## Abstract

**OBJECTIVES:**

The coronavirus disease 2019 (COVID-19) pandemic has negatively affected every aspect of medical care. However, information regarding the impact of the pandemic on cancer screening is lacking. This study aimed to explore cancer screening changes by geographic region before and during the pandemic in Korea.

**METHODS:**

Korean National Cancer Screening Survey data for 2019 and 2020 were used. Changes in the screening rate before and during the COVID-19 pandemic were calculated by subtracting the rate in 2020 from the rate in 2019. Multivariate logistic regression analyses examined the differences in screening rates at the national and 16 provincial levels before and after the COVID-19 outbreak.

**RESULTS:**

The 1-year screening rates for the four types of cancer decreased during the pandemic (stomach cancer: -5.1, colorectal cancer: -3.8, breast cancer: -2.5, cervical cancer: -1.5%p). In metropolitan areas, the odds of undergoing screening tests during the pandemic were significantly lower than before the pandemic for stomach (adjusted odds ratio [aOR], 0.66; 95% confidence interval [CI], 0.56 to 0.76), colorectal (aOR, 0.63; 95% CI, 0.50 to 0.79), and breast cancers (aOR, 0.75; 95% CI, 0.60 to 0.94). Furthermore, the likelihood of undergoing stomach cancer screening during the pandemic was significantly lower than before the pandemic in non-metropolitan urban areas (aOR, 0.81; 95% CI, 0.70 to 0.94), while it was higher in rural areas (aOR, 1.54; 95% CI, 1.10 to 2.16).

**CONCLUSIONS:**

Since the COVID-19 pandemic, the cancer screening rate has decreased significantly, especially in large cities. Public health efforts are required to improve cancer screening rates.

## INTRODUCTION

Coronavirus disease 2019 (COVID-19) is caused by severe acute respiratory syndrome coronavirus 2 (SARS-CoV-2) [1]. Since the first COVID-19-positive patient was diagnosed in December 2019, the disease has rapidly spread worldwide. Many countries have imposed social distancing or even countrywide lockdowns to prevent infection and transmission [2-6]. The resulting changes have affected every part of life, including medical care. During the COVID-19 pandemic, some medical services that are considered non-urgent care have been canceled or rescheduled, such as routine pediatric vaccine programs, annual health check-ups, and cancer screening tests [7-9]. In the United States, the COVID-19 pandemic caused a sharp decline in cancer screening from March to May 2020 compared with 2019, specifically, by 90.8% for breast cancer, 79.3% for colorectal cancer, and 63.4% for prostate cancer [10]. In Northern Taiwan, colorectal screening uptake from December 2019 to April 2020 was 88.8%, which was significantly lower than that in the corresponding period of the past 3 years (91.2 to 92.7%) [11]. A systematic review conducted at the end of 2020 [9] revealed a marked decline in cancer screening owing to the enforcement of stay-at-home guidelines. Patients primarily experienced fear and anxiety about being infected and shortages in medical devices or healthcare workers during the pandemic era.

Delays in cancer screening worldwide may increase emergency conditions, diagnoses of later-stage cancers, and delays in effective treatment for patients with newly diagnosed malignancies. These factors may impact future mortality rates and total years of life lost. For example, a recent study estimated the long-term clinical impact of interruption for breast cancer screening in Canada; a 3-month interruption could increase cases diagnosed at advanced stages (310 more) and cancer deaths (110 more) in 2020-2029 [12].

In Korea, the COVID-19 outbreak began on January 20, 2020, after which the number of cases increased [13]. As the number of infected cases rose across the country and varied regionally, social distancing regulations were issued by the Korean government. To reduce pandemic-related healthcare changes, in April 2020, the Korean Cancer Association and the National Cancer Center implemented guidelines for cancer care during the COVID-19 pandemic, including guidelines for cancer screening [14]. These guidelines recommended that cancer screening should not be delayed in healthy patients. However, in areas where the infection was uncontrolled, interruptions of cancer screening were inevitable. This study aimed to explore the potential differences in changes in cancer screening by geographic region before and during the COVID-19 pandemic in Korea.

## MATERIALS AND METHODS

### Data sources and study population

Data were obtained from the Korean National Cancer Screening Survey (KNCSS) database for 2019 and 2020. The KNCSS is a nationally representative annual cross-sectional survey conducted in 2004 by the National Cancer Center [15]. Participants were selected based on resident registration population data using a stratified, multistage, random sampling procedure according to geographic area, age, and gender. Subjects were recruited through door-to-door visits by a professional research agency, and at least 3 attempts were made to contact a resident in each household. The investigators conducted the face-to-face interviews in participants’ homes. The details of sampling methods and procedures have been described in previous studies [15,16].

The survey was conducted among cancer-free men aged 40-74 years and women aged 20-74 years. A total of 4,500 men and 4,600 women who completed the surveys in 2019 and 2020 were included in the final analysis.

### Measures

Using a structured questionnaire, the KNCSS investigated screening for five types of cancer (stomach, liver, colorectal, breast, and cervical cancer) and socio-demographic characteristics, including educational level, household income, marital status, residential area, and type of health insurance. The screening history included stomach cancer in men and women over 40 years of age, colorectal cancer in men and women over 50 years of age, cervical cancer in women over 20 years of age, and breast cancer in women over 40 years. Eligible participants were asked about their screening experiences according to the guidelines of the Korea National Cancer Screening Program (KNCSP) [17]. The major questions included: “Have you ever undergone (cancer type) screening?,” “Which screening method have you experienced?,” “When did you last undergo (cancer type) screening with this method?.”

In the 2020 survey, some questions were added to collect information regarding a plan for cancer screening uptake and reasons why they did not receive screening during the pandemic, including “Did you plan for cancer screening in 2020?,” “Did you have cancer screening as planned?,” “Why did you not have cancer screening?.”

Lifetime screening rates, screening rates according to recommendations, and the screening rates within the last 1 year were calculated for 2019 and 2020. The lifetime screening rate was defined as having undergone a screening test during one’s lifetime. Meanwhile, the screening rate according to recommendations was defined as the number of participants who had undergone screening tests according to the KNCSP procedures and intervals. The KNCSP guidelines recommended the following screening intervals: upper endoscopy or upper gastrointestinal (UGI) series every 2 years for stomach cancer; fecal occult blood test (FOBT) every year or colonoscopy every 10 years for colorectal cancer; mammography every 2 years for breast cancer; a Pap smear every 2 years for cervical cancer; and ultrasonography every 6 months for liver cancer. Due to the small number of respondents who were at high risk for liver cancer (i.e., those aged over 40 years with hepatitis B virus surface antigen positivity, hepatitis C virus antibody positivity, or liver cirrhosis), we excluded liver cancer from the analysis.

### Statistical analysis

Descriptive statistics were used to summarize the data as frequencies and percentages. The changes in the cancer screening rate (percentage point; %p) before and during the COVID-19 pandemic were calculated by subtracting the rate in 2020 from the rate in 2019, without adjustment for the age and gender distribution in each province. The chi-square test was used to test the differences in cancer screening rates between the 2 years surveyed. The analyses were conducted at the national level and stratified by the subnational level (i.e., metropolitan, non-metropolitan urban, and rural areas) and provinces (i.e., 16 provinces). Logistic regression was performed to compare the odds of cancer screening between 2020 and 2019, adjusted for age and gender. Statistical significance was set at a p-value < 0.05. All analyses were performed using Stata version 16 (StataCorp., College Station, TX, USA).

### Ethics statement

This study was approved by the Institutional Review Board of the National Cancer Center, Korea (IRB No. NCC-2019-0233). All study partici pants were provided with a sufficient explanation, and they agreed to participate in the survey through informed consent.

## RESULTS

The socio-demographic characteristics of the study participants in 2019 and 2020 are presented in Table 1. The distribution of respondents’ socio-demographic characteristics was similar between the 2 years, except that there was a statistically significant difference in the place of residence and type of health insurance.

### Change in cancer screening rates for the 4 types of cancer

The screening rates for the four types of cancer in 2019 and 2020 were compared (Table 2). The lifetime screening rates significantly increased for all four types of cancer, with the screening rate increasing by 3.3%p for stomach cancer and cervical cancer and 5.3%p and 6.0%p for breast cancer and colorectal cancer, respectively.

In contrast, the screening rates according to recommendations were stable. However, in an analysis of the specific screening methods, those who underwent UGI series for stomach cancer screening and FOBT for colorectal cancer screening decreased significantly, whereas those who underwent colonoscopy for colorectal cancer screening increased significantly.

To compare the changes in the screening rate before and during the COVID-19 pandemic, we compared the screening rate within the last 1 year between 2019 and 2020. Interestingly, compared to 2019, the screening rates within the last 1 year of 2020 fell for all four types of cancer. Specifically, the decrease was the largest in stomach cancer screening (-5.1%p), followed by colorectal cancer screening (-3.8%p). The decreases changes in breast cancer and cervical cancer screening were -2.5%p and -1.5%p, respectively, but these decreases were not statistically significant.

### Change in the cancer screening rates by geographical region

The screening rates within the last 1 year in 2019 and 2020 were analyzed by region (Table 3). In metropolitan areas, a significant decrease in the odds of screening was found for stomach, colorectal, and breast cancers. Specifically, after adjusting for age and gender, the odds of screening decreased by 34% for stomach cancer (adjusted odds ratio [aOR], 0.66; 95% confidence interval [CI], 0.56 to 0.76), 37% for colorectal cancer (aOR, 0.63; 95% CI, 0.50 to 0.79), and 25% for breast cancer (aOR, 0.75; 95% CI, 0.60 to 0.94). A significant decrease in the odds of stomach cancer screening was also found in participants living in non-metropolitan urban areas (aOR, 0.81; 95% CI, 0.70 to 0.94), whereas this screening increased significantly in rural areas (aOR, 1.54; 95% CI, 1.10 to 2.16). There was no significant change in the odds of cervical cancer screening in any of the regions.

**Figure 1.**
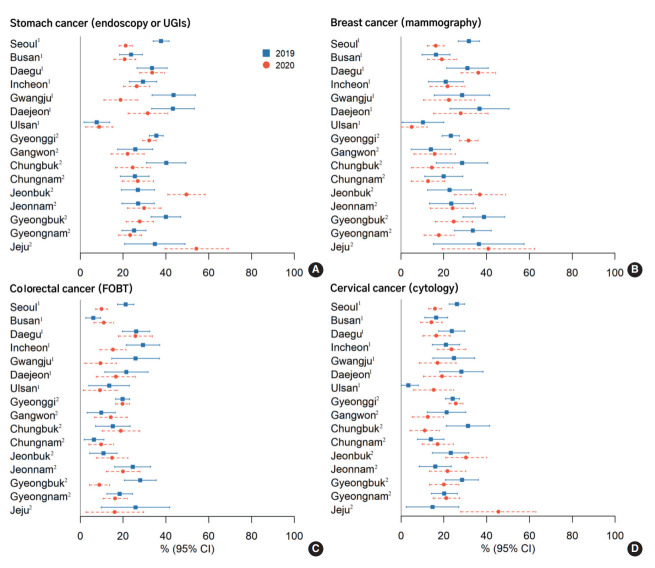
Geographic variability in cancer screening rates for stomach (A), breast (B), colorectal (C), and cervical (D) cancers, by survey year (KNCSS 2019-2020). Age and gender distribution by each province were not considered when analyzing the changes in cancer screening rates. KNCSS, Korean National Cancer Screening Survey, UGI, upper gastrointestinal; FOBT, fecal occult blood test. ^1^Metropolitan provinces. ^2^Provinces that include both non-metropolitan urban and rural areas.

Figure 1 and Supplementary Material 1 show regional variations in cancer screening uptake before and during the COVID-19 pandemic. Ten of 16 provinces (62.5%) had decreased stomach cancer screening rates. Gwangju Province showed the largest decrease, while Jeonbuk Province showed the largest increase in stomach cancer screening rates.

The screening rates for the remaining cancer types also decreased in 9 out of 16 provinces (56.3%) for colorectal cancer, 8 provinces for breast cancer (50.0%), and 6 provinces for cervical cancer (37.5%). Of particular note, the screening rates in Seoul, Gwangju, Daejeon, and Gyeongbuk decreased for all cancer types.

Table 4 presents reasons for not having cancer screening during the COVID-19 pandemic. The most frequent reasons were related to “no symptoms and planning to screen after COVID-19”. Notably, worrying about being infected with the virus accounted for 35.9% among participants living in metropolitan areas, followed by non-metropolitan urban residents (32.2%) and rural residents (23.9%). Other reasons included “did not receive an invitation,” “no one recommends getting a screening,” and “concern about the cost of the screening test.”

## DISCUSSION

Our study was conducted to estimate the changes in cancer screening rates before and during the COVID-19 pandemic using nationally representative surveys. The screening rate within the last year showed the largest decrease for stomach cancer, followed by colorectal cancer screening, while breast cancer and cervical cancer screening rates showed lower magnitudes of decrease. In particular, the effects of COVID-19 have been associated with screening in different regions. In metropolitan areas, significant decreases in screening were observed for stomach cancer, colorectal cancer, and breast cancer. Stomach cancer screening also showed a significant decrease in non-metropolitan urban areas and an increase in rural areas.

As expected, cancer screening rates decreased after the COVID-19 outbreak compared with the pre-pandemic period. Previous studies indicated that screening rates according to recommendations have consistently increased, with annual growth rates of 4.2% for stomach cancer, 3.0% for colorectal cancer, 3.7% for breast cancer, and 1.3% for cervical cancer, from 2004 to 2013 [16]. The period from 2014 to 2018 witnessed non-significant changes in cancer screening rates [18]. The decreases in cancer screening rates in our study; especially the significant decreases for screening within the last year, are believed to have been affected by the COVID-19 pandemic in a different pattern than before. However, these changes were quite small when compared to recent reports from other countries [9-11,19]. All screening services were suspended in the United Kingdom, and referrals for cancer dropped dramatically by 75% within just 4 months after the pandemic started [19]. In Australia, 145,000 fewer mammograms were conducted between January and June 2020, compared to the same period 2 years prior [20]. In the United States, cancer screening decreased sharply from March to May 2020, compared with the same months in 2019, by 90.8% for breast cancer and 79.3% for colorectal cancer [10]. There are several possible explanations for these discrepancies. First, the spread of COVID-19 and responses to the pandemic have been highly heterogeneous across regions of the world. In the first 6 months of 2020, the United Kingdom recorded over 21,000 COVID-19 cases, while the United States reached more than 2,700,000 cases [21]. At the same time, the number of confirmed cases in Australia and Korea was 7,800 and 12,800, respectively [21]. A full nationwide lockdown was enforced from March to May 2020, in the United Kingdom and Australia [22]. By contrast, the Korean government has not enforced a lockdown policy on the entire country; instead, a social distancing system was implemented with 4 levels (levels 1 to 4 [highest]) depending on the COVID-19 status of each region [23]. Second, despite the threat of the COVID‐19 pandemic, Korea is one of the few countries where cancer care is ongoing. In April 2020, the Korean Cancer Association and National Cancer Center issued guidelines for cancer care during COVID-19, including specific recommendations for surgery, chemotherapy, radiotherapy, pediatric oncology, and cancer screening [14]. Third, Korea has a comprehensive and universal healthcare system [24]. Since all residents receive the same medical services and benefits provided by the National Health Insurance Service at minimum or no cost, the decline in people’s economic circumstances during the pandemic would not have much impact on medical use. Moreover, the KNCSP is still underway for the entire population, even during COVID-19.

Nevertheless, during COVID-19, crowded areas, such as metropolitan areas, showed a significant decrease in screening rates compared with non-metropolitan urban and rural areas. This finding is similar to that in the United States [10], where the decrease in screening was found associated with different geographic regions; specifically, areas reporting early and higher surges of COVID-19 cases experienced larger declines in cancer examinations. At the start of the survey in 2020 (August 5, 2020), 14,456 confirmed COVID-19 cases, including 302 deaths, had been reported in Korea [25]. Metropolitan areas, including Seoul, Busan, Daegu, Incheon, Gwangju, Daejeon, and Ulsan, showed 8,959 confirmed cases, which accounted for roughly two-thirds of cases. In addition, Seoul and Gwangju Metropolitan Cities, where the first COVID-19 cases were detected in the community, showed decreases in almost all cancer screening types. Besides the high prevalence of COVID-19, the fear of infection has also been proposed as a cause of these decreases. According to a previous study conducted in Korea, there was a significant upward trend in the rates of nonparticipation in pre-scheduled health check-ups when the fear of COVID-19 exceeded that of lung cancer [26]. Our study found that 35.9% of those who did not undergo cancer examinations in metropolitan areas were concerned about COVID-19 infection, which was higher than the proportions of 32.2% in non-metropolitan urban areas and 23.9% in rural counties. The combination of increased COVID-19 confirmed cases, a strong social distancing policy, and fear of being infected by COVID-19 may have led to these decreases in cancer screening rates, especially in metropolitan areas. Therefore, if COVID-19 is not well controlled and risk communication strategies are not well established, preventive services such as cancer screening will be affected more severely. Furthermore, the reduction in cancer screening participation negatively affected cancer diagnoses after the COVID-19 pandemic. While the pandemic is still not under control, interventions to enhance the awareness of the public and their attendance of cancer screening, as well as a sustainable and flexible outreach system, could be considered in the community as preparation for future pandemics.

This study had some limitations. First, all information was retrospectively collected through a self-reported questionnaire; thus, recall bias might have occurred, albeit to a relatively small degree, as we investigated information on recent health-screening experiences. Second, since the COVID-19 outbreak began in early 2020, we could only compare screening rates over the past year. Most cancer screening guidelines recommend screening every 2 years. Therefore, this study has limitations in sufficiently observing changes in screening rates according to recommendations before and after COVID-19. Despite these limitations, to the best of our knowledge, this is the first population-based study to demonstrate that deficits in both opportunistic and organized cancer screening rates differed by geographic region during the COVID-19 pandemic in Korea.

In conclusion, early detection through screening can reduce the cancer burden; however, screening rates have fallen during the COVID-19 pandemic. The largest decrease in screening rates was found in metropolitan areas, which is believed to be associated with the COVID-19 pandemic. This study shows that a pandemic can cause problems in healthcare, such as delaying the early detection of cancer. Therefore, actions are required to mitigate the potential negative impact of COVID-19 on cancer prevention. After the pandemic, public health efforts must resume to increase screening rates, especially in metropolitan areas.

## Figures and Tables

**Table 1.** Socio-demographic characteristics of study participants from the Korean National Cancer Screening Survey, 2019-2020

Characteristics	Survey year, %		p-value
	2019	2020	
Total no. of respondents (n)	4,500	4,600	
Gender			0.730
Men	38.8	39.1	
Women	61.2	60.9	
Age (yr)			0.788
20-29	11.1	10.9	
30-39	10.2	9.8	
40-49	24.8	24.3	
50-59	25.5	25.2	
60-69	18.0	19.1	
70-74	10.4	10.7	
Education			0.342
Lower secondary school	13.0	11.8	
High school	46.2	47.4	
University/College	40.5	40.4	
Postgraduate	0.4	0.4	
Monthly household income (US$)^1^			0.297
≤1,999	7.5	8.4	
2,000-3,999	34.6	34.4	
≥4,000	57.9	57.2	
Residential area			0.004
Metropolitan	44.5	47.4	
Non-metropolitan urban	45.7	44.3	
Rural	9.8	8.3	
Provinces			<0.001
Seoul	19.4	18.9	
Busan	6.9	6.7	
Daegu	4.8	6.8	
Incheon	5.7	5.6	
Gwangju	2.7	2.7	
Daejeon	2.8	2.7	
Ulsan	2.2	2.2	
Gyeonggi	24.8	24.7	
Gangwon	3.0	3.0	
Chungbuk	3.1	3.0	
Chungnam	4.5	3.9	
Jeonbuk	3.5	3.4	
Jeonam	3.6	3.5	
Gyeongbuk	5.2	5.0	
Gyeongnam	6.4	6.3	
Jeju	1.3	1.2	
Health insurance type			<0.001
National Health Insurance	98.6	99.5	
Medical Aid Program	1.4	0.5	

1 1 US$=1,000 Korean won.

**Table 2.** Changes in cancer screening rates for 4 types of cancer (KNCSS 2019-2020)

Variables	Survey year, %		Change (%p)	p-value
	2019	2020		
Lifetime screening rate^1^				
Stomach	83.4	86.7	3.3	<0.001
Colorectal	77.2	83.2	6.0	<0.001
Breast	81.9	87.2	5.3	<0.001
Cervical	61.1	64.4	3.3	0.012
Screening rate according to recommendations^2^				
Stomach^3^	70.8	68.7	-2.1	0.063
Upper endoscopy	62.1	61.2	-0.9	0.401
UGI series	25.8	23.2	-2.5	0.010
Colorectal^4^	62.6	64.3	1.7	0.201
FOBT	19.1	15.3	-3.8	<0.001
Colonoscopy	51.5	57.5	6.0	<0.001
Breast^5^	62.3	63.5	1.2	0.458
Cervical^6^	51.0	50.6	-0.4	0.751
Screening rate within 1 yr^7^				
Stomach^8^	32.7	27.6	-5.1	<0.001
Upper endoscopy	27.0	24.2	-2.8	0.006
UGI series	10.0	7.8	-2.2	0.001
Colorectal^9^	19.1	15.3	-3.8	<0.001
Breast^10^	26.3	23.8	-2.5	0.083
Cervical^11^	22.2	20.7	-1.5	0.173

KNCSS, Korean National Cancer Screening Survey; UGI, upper gastrointestinal; FOBT, fecal occult blood test.

1 The lifetime screening rate was defined as the proportion of target-aged respondents who had ever undergone the screening test(s).

2 The screening rate according to recommendations was defined as the proportion of respondents who had fulfilled the screening recommendation criteria among the respondents in the targeted age group for relevant cancer.

3 Respondents who had last undergone upper endoscopy or UGI series screening within 2 years, among men and women aged ≥ 40 years.

4 Respondents who had last undergone screening with colonoscopy or FOBT within 10 years and 1 year, respectively, among men and women aged ≥ 50 years.

5 Respondents who had last undergone screening with mammography within 2 years, among women aged ≥ 40 years.

6 Respondents who had last undergone screening with conventional cytology within 2 years among women aged ≥ 20 years.

7 The screening rate within 1 year was defined as the proportion of respondents who underwent the latest screening test within 1 year among target-aged respondents for relevant cancer.

8 Respondents who had the latest upper endoscopy or UGI series screening within 1 year, among men and women aged ≥ 40 years.

9 Respondents who had the latest FOBT within 1 year, among men and women aged ≥ 50 years.

10 Respondents who had the latest mammography within 1 year, among women aged ≥ 40 years.

11 Respondents who had the latest conventional cytology within 1 year, among women aged ≥ 20 years.

**Table 3.** Multivariable logistic regression of changes in cancer screening within the last year before and after the COVID-19 pandemic, by geographic region^1^

Cancerrs	Metropolitan (n=4,180)		Non-metropolitan urban (n=4,096)		Rural (n=824)	
Stomach						
2019	33.1	1.00 (reference)	34.9	1.00 (reference)	22.5	1.00 (reference)
2020	24.5	0.66 (0.56, 0.76)^***^	30.3	0.81 (0.70, 0.94)^**^	30.1	1.54 (1.10, 2.16)^*^
Colorectal						
2019	20.1	1.00 (reference)	19.4	1.00 (reference)	15.0	1.00 (reference)
2020	13.6	0.63 (0.50, 0.79)^***^	18.4	0.93 (0.75, 1.16)	10.5	0.65 (0.40, 1.07)
Breast						
2019	26.9	1.00 (reference)	26.4	1.00 (reference)	23.2	1.00 (reference)
2020	21.6	0.75 (0.60, 0.94)^*^	27.4	1.05 (0.84, 1.31)	19.0	0.80 (0.48, 1.33)
Cervical						
2019	21.6	1.00 (reference)	24.2	1.00 (reference)	14.9	1.00 (reference)
2020	18.8	0.84 (0.69, 1.01)	23.4	0.95 (0.79, 1.15)	17.6	1.33 (0.80, 2.21)

Values are presented as percentage or adjusted odds ratio (95% confidence interval). COVID-19, coronavirus disease 2019.

1 Adjusted by age and gender.

* p<0.05,

** p<0.01,

*** p<0.001.

**Table 4.** Reasons for not having cancer screening during the coronavirus disease 2019 (COVID-19) outbreak in 2020 (n=3,201)

Variables	Metropolitan (n=1,507)	Non-metropolitan urban (n=1,406)	Rural (n=288)
Because I have no symptoms, I will have screening after the COVID-19 pandemic	574 (38.1)	610 (43.4)	141 (49.0)
I am worried about becoming infected with COVID-19	540 (35.9)	452 (32.2)	69 (23.9)
I did not receive any invitation	227 (15.1)	227 (16.1)	41 (14.2)
No one recommended me to get screened	121 (8.0)	86 (6.1)	16 (5.6)
Costs	20 (1.3)	8 (0.6)	0 (0.0)
I have a fever or respiratory symptoms	8 (0.5)	9 (0.6)	0 (0.0)
Others	17 (1.1)	14 (1.0)	21 (7.3)

Values are presented as number (%).

## SUPPLEMENTARY MATERIALS

Supplementary Material 1. COVID-19 confirmed cases and cancer screening rates for stomach, colorectal, breast, and cervical cancers in 2019 and 2020, by region
