# Supplementary material for: Changes in cancer screening before and during COVID‐19: findings from the Korean National Cancer Screening Survey 2019 and 2020
Source: Epidemiol Health. 2022 May 30;44:e2022051. doi: 10.4178/epih.e2022051 (PMC9754916; doi:10.4178/epih.e2022051)
Supplement: Supplementary Material 1. — COVID-19 confirmed cases and cancer screening rates for stomach, colorectal, breast, and cervical cancers in 2019 and 2020, by region [file epih-44-e2022051-suppl.docx]

Supplementary Material 1. COVID-19 confirmed cases and cancer screening rates for stomach, colorectal, breast, and cervical cancers in 2019 and 2020, by region

| Province | Total COVID-19 confirmed cases^1^ | Stomach cancer screening  (Endoscopy or UGIs) | | | Colorectal cancer screening (FOBT) | | | | | | Breast cancer screening  (Mammography) | | | | Cervical cancer screening (Cytology) | | | | |  |
| --- | --- | --- | --- | --- | --- | --- | --- | --- | --- | --- | --- | --- | --- | --- | --- | --- | --- | --- | --- | --- |
|  |  | Year  % (95%CI) | | *p*-value | Year  % (95%CI) | | | | *p*-value | | Year  % (95%CI) | | | *p*-value | Year  % (95%CI) | | | *p*-value | |  |
|  |  | 2019 | 2020 |  | 2019 | | 2020 | |  | | 2019 | 2020 | |  | 2019 | 2020 | |  | |  |
| Seoul^2^ | 1,280 | 37.7 (34.0,41.5) | 21.3 (18.1,24.4) | <0.01 | | 21.2 (17.4,25.0) | | 10.0 (7.2,12.8) | | <0.01 | 31.8 (26.8,36.7) | | 16.3 (12.3,20.2) | <0.01 | 26.1 (22.4,29.7) | | 15.9 (12.9,19.0) | | <0.01 | |
| Busan^2^ | 132 | 23.7 (18.4,29.0) | 20.8 (15.7,25.9) | 0.44 | | 6.1 (2.6,9.6) | | 11.0 (6.4,15.6) | | 0.10 | 16.4 (9.9,22.9) | | 19.2 (12.4,26.1) | 0.55 | 16.4 (11.1,21.7) | | 14.2 (9.2,19.2) | | 0.55 | |
| Daegu^2^ | 6,881 | 33.5 (26.5,40.6) | 33.6 (27.8,39.3) | 0.99 | | 26.1 (19.7,32.5) | | 25.8 (17.9,33.7) | | 0.96 | 31.1 (21.4,40.8) | | 36.2 (28.1,44.3) | 0.43 | 23.7 (17.6,29.8) | | 16.5 (10.2,22.9) | | 0.12 | |
| Incheon^1^ | 305 | 29.3 (22.9,35.7) | 26.4 (20.2,32.5) | 0.51 | | 29.3 (21.5,37.1) | | 15.3 (9.2,21.4) | | 0.01 | 21.0 (12.9,29.1) | | 21.8 (13.6,29.9) | 0.89 | 21.0 (14.6,27.4) | | 23.6 (16.9,30.3) | | 0.59 | |
| Gwangju^1^ | 180 | 43.6 (33.5,53.8) | 18.9 (11.0,26.9) | <0.01 | | 25.8 (14.7,36.9) | | 9.5 (2.1,16.9) | | 0.02 | 28.6 (15.6,41.5) | | 22.4 (10.5,34.4) | 0.49 | 24.7 (14.9,34.4) | | 17.1 (8.5,25.7) | | 0.25 | |
| Daejeon^2^ | 147 | 43.3 (33.3,53.3) | 31.6 (22.3,40.9) | 0.09 | | 21.5 (11.4,31.7) | | 16.7 (7.5,25.8) | | 0.48 | 36.7 (22.9,50.5) | | 28.0 (15.3,40.7) | 0.35 | 28.2 (18.1,38.3) | | 19.2 (10.4,28.1) | | 0.19 | |
| Ulsan^2^ | 34 | 7.7 (1.7,13.7) | 8.9 (2.5,15.2) | 0.79 | | 13.5 (4.0,22.9) | | 9.3 (1.4,17.2) | | 0.49 | 10.3 (0.5,20.1) | | 5.1 (0.0,12.3) | 0.4 | 3.4 (0.0,8.1) | | 15.3 (5.9,24.6) | | 0.03 | |
| Gyeonggi^3^ | 1,149 | 35.5 (32.3,38.7) | 32.3 (29.2,35.4) | 0.16 | | 19.8 (16.5,23.0) | | 19.8 (16.5,23.0) | | 0.99 | 23.3 (19.3,27.3) | | 31.7 (27.3,36.0) | 0.01 | 24.1 (20.9,27.3) | | 25.6 (22.4,28.9) | | 0.52 | |
| Gangwon^3^ | 53 | 25.7 (17.5,33.8) | 22.1 (14.4,29.9) | 0.53 | | 9.8 (3.2,16.3) | | 14.3 (6.7,21.9) | | 0.37 | 14.0 (4.8,23.2) | | 15.8 (6.1,25.4) | 0.79 | 21.3 (12.2,30.3) | | 12.5 (5.2,19.8) | | 0.14 | |
| Chungbuk^3^ | 62 | 40.2 (31.0,49.3) | 24.5 (16.4,32.7) | 0.01 | | 15.2 (7.2,23.2) | | 19.0 (10.2,27.8) | | 0.53 | 28.6 (16.5,40.6) | | 14.5 (5.0,24.1) | 0.07 | 31.3 (21.2,41.4) | | 11.1 (4.2,18.0) | | <0.01 | |
| Chungnam^3^ | 159 | 25.5 (18.7,32.2) | 26.9 (19.6,34.2) | 0.78 | | 6.4 (1.8,11.0) | | 9.8 (4.0,15.6) | | 0.36 | 20.0 (11.1,28.9) | | 12.7 (4.8,20.5) | 0.11 | 13.9 (7.7,20.1) | | 17.1 (9.9,24.4) | | 0.17 | |
| Jeonbuk^3^ | 182 | 26.9 (19.2,34.6) | 49.6 (40.9,58.3) | <0.01 | | 10.8 (4.4,17.1) | | 14.9 (7.6,22.2) | | 0.40 | 22.7 (12.4,33.0) | | 36.9 (25.0,48.9) | 0.08 | 23.2 (14.6,31.7) | | 30.4 (20.9,40.0) | | 0.26 | |
| Jeonnam^3^ | 17 | 27.0 (19.5,34.5) | 29.9 (22.0,37.7) | 0.06 | | 24.5 (16.1,32.9) | | 20.0 (12.1,27.9) | | 0.44 | 23.5 (13.3,33.8) | | 24.2 (13.7,34.8) | 0.92 | 16.0 (8.5,23.4) | | 21.7 (13.2,30.3) | | 0.31 | |
| Gyeongbuk^3^ | 1,372 | 40.0 (33.1,46.9) | 27.8 (21.5,34.2) | 0.01 | | 28.0 (20.6,35.4) | | 9.0 (4.3,13.7) | | <0.01 | 38.8 (29.0,48.5) | | 24.7 (16.1,33.4) | 0.04 | 28.5 (20.8,36.1) | | 20.1 (13.3,27.0) | | 0.11 | |
| Gyeongnam^3^ | 111 | 25.1 (19.5,30.7) | 23.3 (17.9,28.7) | 0.65 | | 18.4 (12.4,24.4) | | 16.3 (10.6,21.9) | | 0.61 | 33.6 (25.0,42.2) | | 17.8 (10.8,24.8) | 0.01 | 20.2 (14.2,26.3) | | 21.2 (15.0,27.4) | | 0.83 | |
| Jeju^3^ | 11 | 34.8 (20.7,48.9) | 54.3 (39.6,69.1) | 0.06 | | 25.8 (9.8,41.8) | | 16.1 (2.7,29.6) | | 0.35 | 36.4 (15.2,57.5) | | 40.9 (19.3,62.5) | 0.76 | 14.7 (2.4,27.0) | | 45.5 (27.9,63.0) | | 0.010 | |

^1^ From January 3^rd^ ~ August 5th, 2020; ^2^Metropolitan provinces; ^3^Provinces that include both urban and rural areas

95%CI: 95% Confidence Interval; *^*^ p < 0.05, ^**^ p < 0.01, ^***^ p < 0.001*
